# Exploring a method for extracting concerns of multiple breast cancer patients in the domain of patient narratives using BERT and its optimization by domain adaptation using masked language modeling

**DOI:** 10.1371/journal.pone.0305496

**Published:** 2024-09-06

**Authors:** Satoshi Watabe, Tomomi Watanabe, Shuntaro Yada, Eiji Aramaki, Hiroshi Yajima, Hayato Kizaki, Satoko Hori

**Affiliations:** 1 Division of Drug Informatics, Keio University Faculty of Pharmacy, Tokyo, Japan; 2 Nara Institute of Science and Technology, Nara, Japan; 3 Mediaid Corporation, Tokyo, Japan; Indian Institute of Technology Patna, INDIA

## Abstract

Narratives posted on the internet by patients contain a vast amount of information about various concerns. This study aimed to extract multiple concerns from interviews with breast cancer patients using the natural language processing (NLP) model bidirectional encoder representations from transformers (BERT). A total of 508 interview transcriptions of breast cancer patients written in Japanese were labeled with five types of concern labels: "treatment," "physical," "psychological," "work/financial," and "family/friends." The labeled texts were used to create a multi-label classifier by fine-tuning a pre-trained BERT model. Prior to fine-tuning, we also created several classifiers with domain adaptation using (1) breast cancer patients’ blog articles and (2) breast cancer patients’ interview transcriptions. The performance of the classifiers was evaluated in terms of precision through 5-fold cross-validation. The multi-label classifiers with only fine-tuning had precision values of over 0.80 for "physical" and "work/financial" out of the five concerns. On the other hand, precision for "treatment" was low at approximately 0.25. However, for the classifiers using domain adaptation, the precision of this label took a range of 0.40–0.51, with some cases improving by more than 0.2. This study showed combining domain adaptation with a multi-label classifier on target data made it possible to efficiently extract multiple concerns from interviews.

## Introduction

Breast cancer is the most commonly diagnosed cancer among women and its incidence is rising worldwide [1–3]. Breast cancer treatment tends to be prolonged, and the increasing incidence of the disease in the 30s and 40s, a time of many potentially major life events, may cause various problems for patients, including employment issues, financial pressures, and psychosocial stress [4]. The onset of breast cancer has a significant impact on the patients’ work and employment prospects, but few patients consult with medical professionals about such issues, and the actual situation is often not well understood [5]. In addition, patients also have physical concerns that are unique to breast cancer, such as breast reconstruction after surgery, or fertility problems caused by drugs and radiation therapy, but these concerns are poorly shared with medical professionals [6–8].

With the development of internet services in recent years, many patients are using social media, such as Twitter, to spread information [9, 10]. Such patients’ narratives may provide information about their needs that they do not tell medical professionals, and they are also a valuable resource for other patients to find information they want, which may improve their quality of life. In order to explore ways to utilize such narratives, qualitative research methods have been used [11–13]. However, the sheer volume of information on the internet makes it difficult to analyze, and therefore methods using NLP have been proposed as a way to process these large amounts of data [14].

In NLP approach, both unsupervised and supervised machine learning methods are being used worldwide to analyze patients’ stories. Many unsupervised machine learning approaches have been studied for content analysis, using topic models such as Latent Dirichlet Allocation (LDA) [15]. In supervised learning approaches, on the other hand, classical algorithms such as support vector machine (SVM) and naive bayes classifier (NB), and more recently, complicated deep learning frameworks such as long short-term memory (LSTM) [16] and BERT [17] are often used [18–21]. In supervised learning, tasks such as classification and unique expression extraction (NER) are used to extract useful knowledge from records of patients helping each other in the community [22, 23]. However, few studies have dealt primarily with patients’ concerns.

In this situation, we have recently reported developing BERT-based classifier to automatically classify multiple concerns from blog posts by patients with breast cancer [24]. Although some improvements are needed in terms of the performance of the model, our previous study showed that the NLP model can extract multiple concerns. Since breast cancer patients have multiple concerns and their concerns may change over time, the creation of such a multi-label classifier is a stepping stone to creating a higher-quality information delivery system for individual patients. In recent years, however, the main platforms for patients to share and obtain information on the internet have become Facebook, YouTube, podcasts, and other platforms, rather than blogs [25, 26]. The major difference from blogs is that patients’ thoughts are often expressed in the form of interviews, and the spoken rather than the written word is becoming the main form of communication. In Japanese conversation, there is a great variety of slurs, mispronunciations, word-chokes, and onomatopoeia, and the gap between the written and spoken language is greater than in English. Applying NLP models to text from speech transcripts therefore has the potential to yield findings that differ from those of previous studies, and such attempts could be helpful for implementing better information-providing systems in the future, especially in non-English-speaking countries.

Therefore, this study aimed to examine the applicability of the BERT-based classifier proposed in the previous study to breast cancer patients’ interviews and also explore methods to improve the performance of the classifier for patients’ narratives. Our results indicate that the "physical" and "work/financial" concerns of breast cancer patients can be efficiently extracted using BERT-based methods not only from text sources, as previously reported, but also from spoken materials such as interview transcriptions. Furthermore, we show here that domain adaptation using MLM is effective for improving performance in the patient narrative domain, and this opens up potential future applications utilizing a variety of unstructured patient-generated data such as narrative text. These findings suggests that the application of natural language processing to a broader range of source materials than have been employed to date, including speech transcripts, will be helpful to improve the provision of personalized advice to patients.

## Materials and methods

### Overview

**Fig 1:**
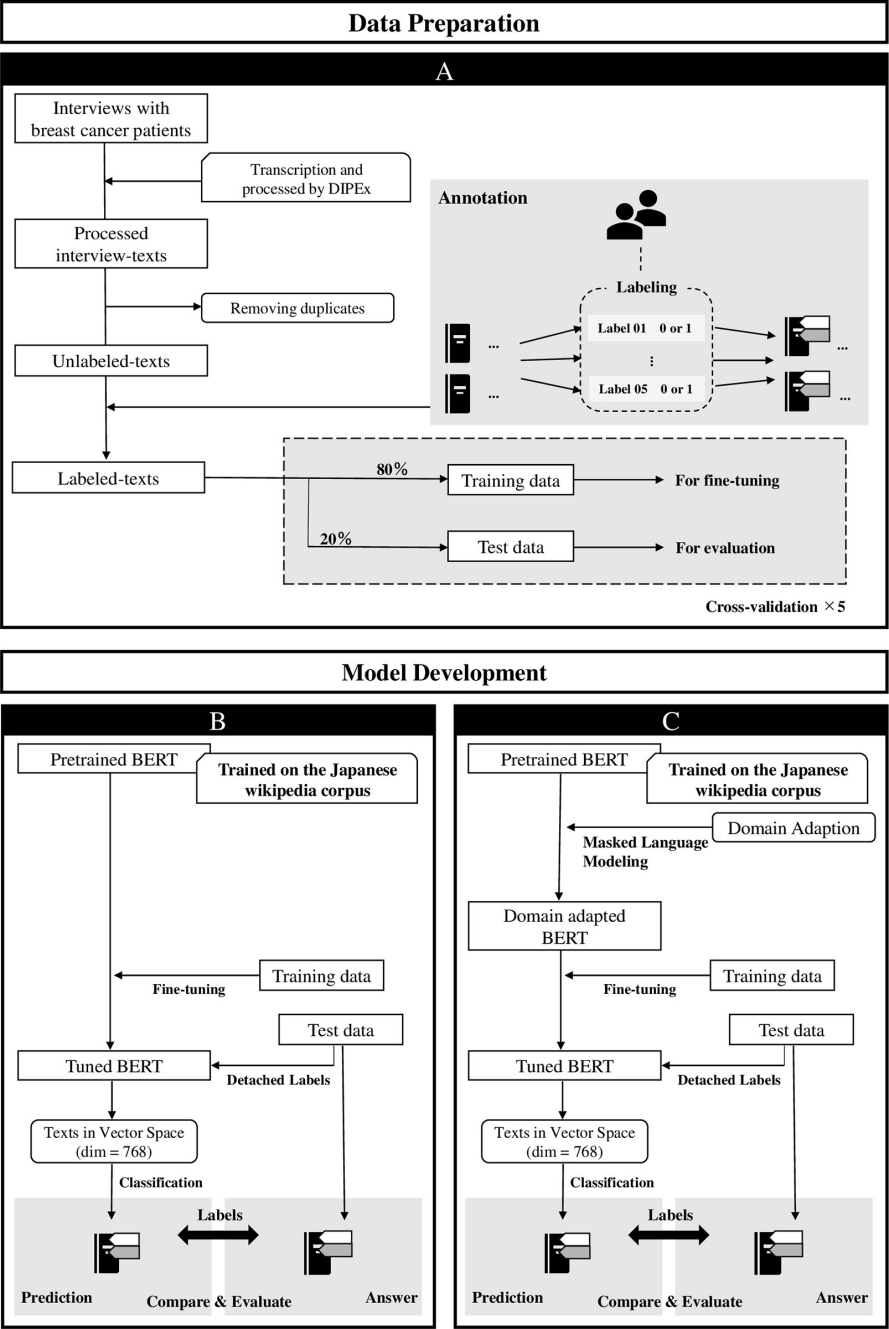
Procedures for data pre-processing and model development. A: Multiple five concern l Multiple five concern labels were assigned to each text. Labeled texts were split into two datasets in a ratio of 4:1: training data (406), and test data (102). B: The classifier was built by fine-tuning the pre-trained BERT model. Training data in the previous section was used. C: The method of fine-tuning is the same as in B, except that the BERT model was additionally trained using masked language modeling on domain-specific data before fine-tuning. We prepared multi datasets for this. A detailed description of each dataset is given in the “Data collection” section and Table 1.

This section describes the dataset used in the study, the methodology for developing the classifier, and the metrics for evaluating it. Fig 1 shows the flow of data extraction and model development. First, text excerpts from interviews with breast cancer patients were collected, and the texts were annotated according to predetermined guidelines. In addition, the annotated text was segmented for model development using 5-fold cross-validation (Fig 1A).

**Table 1:** Overview of classifier development.

Model	Data for MLM	Fine-tuning data
**Fine-tuning only**		
	**×**	DIPEx (P)
	**×**	DIPEx (P)+LifePalette
**Fine-tuning +MLM**		
	DIPEx raw	DIPEx (P)
	DIPEx (P)	DIPEx (P)
	LifePalette	DIPEx (P)
	DIPEx (P)+LifePalette	DIPEx (P)

DIPEx (P): Interview transcripts broken down by each theme

LifePalette: Breast cancer blog articles on the patient web community

DIPEx raw: Unprocessed interview transcriptions

Then, we built the classifier from processed texts dealing with patient concerns. In this study, two main ways of model development were tried. Fig 1B and 1C shows the model development methodology. In the first method, a pre-trained NLP model was fine-tuned directly with the target data. As another method, in an attempt to improve the performance of the model, we tested additional model training to adapt the model to the patients’ narratives domain prior to fine-tuning. For the data used in the domain adaptation here, there are several combinations, which are presented in more detail in Table 1 below.

### Data collection

The patients’ narratives data were collected from interview transcripts of patients with breast cancer registered in Database of Individual Patient Experiences-Japan (hereinafter, called DIPEx) [27]. DIPEx divides the interview transcriptions into sections for each theme, such as "onset of illness" and "treatment", and posts the extracted processed texts on its website. Each process is conducted by accredited researchers based on qualitative research methods established by the University of Oxford [28]. In this study, interview text data generated from interviews with 52 breast cancer patients conducted from January 2008 to October 2018 were provided with the approval of DIPEx, of which 508 were included, after excluding duplicates (hereafter, this data is referred to as processed DIPEx, (DIPEx(P)). In addition, to explore ways to improve classifier performance, we used Japanese blog articles in the patient web community LifePalette [29], one of the most active internet patient communities in Japan, along with texts from DIPEx in a domain adaptation test.

### Ethical approval

This study was approved by the ethics committee of the Keio University Faculty of Pharmacy (approval No 220311–1, 221205–1). All procedures were performed in accordance with the Ethical Guidelines for Medical and Health Research Involving Human Subjects (set by the Ministry of Education, Culture, Sports, Science and Technology and the Ministry of Health, Labour and Welfare in Japan) and the Declaration of Helsinki and its later amendments. Informed consent for this study was waived due to its retrospective observational design. DIPEx data were provided through data sharing, a system for utilizing narrative data for research and education, and consent for this was obtained from the research participants at the time when the interviews were recorded. Consent to use the data from Life Palette for research purposes was obtained from users at the time of registration. Although non-textual information such as age at diagnosis and gender information is present in both data sources, we had consent to utilize only textual data for research purposes in this study.

### Data pre-processing

In this study, we defined concern labels as expressions that indicate what concerns are described in the interview transcriptions. The annotation guidelines describing the criteria for assigning labels were used in accordance with previous studies [24] (S1 Table). Labels are assigned if there are expressions of cancer-related concerns in the text. Multiple labels were allowed if more than one description of distress corresponded to a single text. In these guidelines, five labels, “treatment,” “physical,” “psychological,” “work/financial,” and “family/friends,” were established based on the Shizuoka Classification, a method of classifying cancer patients’ problems established from a previous survey [30]. Based on the annotation guidelines, two researchers (SW and TW) assigned labels to 100 randomly selected texts out of a total of 508 texts, 50 each in two sessions. Although the guidelines were thoroughly discussed prior to annotation, the first session was used to establish the annotation method and how to handle the guidelines, and the second session was used to confirm the reliability of the guidelines. The agreement of the assignment results was confirmed using the kappa coefficient, and finally SW assigned labels to all texts.

### Model development

Table 1 shows a list of all classification model development patterns that were tried in this study. To develop the classifier, BERT, a context-aware, large-scale NLP model, was used. BERT is trained through a two-step learning process. The first step is pretraining from scratch on a large text data set, which allows the model to learn generic features common to various tasks and the second step is to fine-tune the pre-trained model for the desired task using a small amount of new data (Fig 1B and 1C).

The model was developed by fine-tuning the pre-trained Japanese BERT model from the Inui/Suzuki Lab at Tohoku University [31]. Hereafter, this pre-trained model-based model will be referred to as J-based BERT. In addition, domain adaptation was tried before fine-tuning in order to explore ways to improve the performance of the model. It is often the case that the characteristics of the data used in fine-tuning are different from those used in pre-training, and in such cases, it is known that additional training with data from the target domain before fine-tuning improves the performance of the model [32, 33].

In this case, the J-based BERT was pre-trained on a large volume of text from the Japanese version of Wikipedia. However, the data used for fine-tuning was interview transcriptions, and the nature of the two types of texts is very different. Furthermore, the small volume of data made it difficult to tune the model for the task. The reason for this is that transcriptions of interviews are costly, because they are created through many processes, including the appropriate treatment for faltering or misspoken words. This makes it difficult to acquire large amounts of data compared to text data available from Twitter, blogs, etc. Domain adaptation is effective when it is difficult to prepare data for such a specific field. In this case, masked language modeling (MLM), one of BERT’s pre-training methods, was used as the domain adaptation method.

This method is self-supervised learning, which means that unlabelled text can also be used for training. Therefore, pre-processed patient interview transcripts and texts from related domains, labelled or unlabelled, may also be used as resources. The amount of relevant data at hand is large compared to the data covered in this study, leading to an exploration of how to make use of unprocessed interview transcripts. Prior research suggests that choosing an MLM-based approach as domain adaptation improves performance in downstream classification tasks [34, 35]. By adding such domain-specific data, domain adaptation allows the weights to be adjusted before fine-tuning the model, thus mitigating the effects of differences in the nature of the data during fine-tuning and pre-training. In this study, MLM was applied based on the pre-trained model tuning method supported by the Huggingface package [36]. The goal of MLM is cross entropy loss in predicting masked tokens. In this study, as in the original paper on BERT, we selected 15% of the input tokens for possible substitution [17]. NVIDIA’s Tesla M10 was used for training, batch size was set to 8, and the number of epochs was set to 30, since there was no difference in the results for larger numbers of epochs. After the MLM was applied, fine-tuning was done. The parameters for fine-tuning were the same as in the previous study [24]: the model consisted of 12 layers of BERT itself and a fully connected layer, the loss function was set to cross-entropy, the batch size to 16, and the learning rate to 10^−5^, and fine-tuning was performed. However, unlike previous studies, the number of epochs was set to 30 and early stopping was implemented with patience. These methods were applied to all five labels for fine-tuning.

Table 1 summarizes all the dataset patterns tested: pre-processed DIPEx interview transcriptions (DIPEx raw), processed DIPEx (P), LifePalette, and DIPEx (P)+LifePalette.

### Task and metrics

Using the test data, a multi-label classification task was performed to predict whether the text contained descriptions corresponding to each concern label. Since the ultimate goal is to apply the constructed model as an information support system for patients with concerns, it is preferable to construct a model with less out-of-place information provision. Therefore, we assessed the performance of the model by focusing on precision for each label. In addition, other evaluation items were F1 score and exact match accuracy, which indicates the percentage of correct prediction of all concern labels. For the evaluation, we used the average of 5-fold cross-validation results. For the evaluation of significant differences in precision scores for each label between models, we employed a two-tailed test with the Bonferroni correction for multiple comparison. The criterion of significance was p < 0.05.

## Results

### Annotated dataset

The average number of words per text in the interview transcriptions was 428.9, the median was 416.0, and the minimum and maximum were 103 and 1054. Texts with 512 words or less that were not caught by BERT’s input limit were 367/508 (72.2% of all texts). Of the remaining 141 texts, 109 (over 75%) were 700 words or less. The number of labels was highest for “physical (212)” and lowest for “work/financial (42)”. The number of labels per text was 0 or 1 for about 80% of the total, making it difficult to assign labels overall. Less than 3% of all texts were assigned three or more labels (Table 2). The label combinations that frequently occurred when there was more than one label assigned were “physical” and “psychological” (35), “treatment” and “physical” (27), and “physical” and “family/friends” (20), which were particularly frequent combinations with “physical”. This was followed by “psychological” and “family/friends” (20), “treatment” and “psychological” (10), while the others were less than 10.

**Table 2:** Statistics and annotation results (n = 508).

	Posts, n (%)
**Labels**	
Treatment	67 (13.2%)
Physical	212 (41.7%)
Psychological	81 (16.0%)
Work/financial	42 (8.3%)
Family/friends	70 (13.8%)
**Number of labels**	
0	154 (30.3%)
1	252 (49.6%)
2	87 (17.2%)
3	14 (2.8%)
4	1 (0.20%)
5	0 (0.0%)

The kappa coefficients were 0.64 for "treatment," 0.73 for "physical," 0.65 for "psychological," 0.83 for "work/financial," and 0.77 for "family/friends," with all labels being in the *substantial* or *almost perfect* category of Landis and Koch.

Table 3 shows excerpts of the statements that supported the assignment of concern labels at the time of annotation, for each label. The original texts in Japanese are also included in S2 Table. Due to the nature of the interview data, most of the descriptions were chronologically in the past.

**Table 3:** Examples of annotation.

Labels	Texts
Treatment	I was a little apprehensive about the breast reconstruction procedure. I was told that because I had undergone radiation therapy, it would be difficult to reconstruct my breast with an artificial one because the skin would not stretch easily.
Physical	My fingernails started showing symptoms. The nails turned black, and even the slightest object hitting them caused severe pain.
Psychological	I cried a lot during the first cancer announcement and I thought, "What will happen to the rest of my family after I die?"
Work/financial	But I guess what worries me is the money (laughs). I’m still getting by now, but every week 25,000 yen goes flying by.
Family/friends	I wondered what would happen to my children and my husband. I really had no choice, I couldn’t help myself. I felt as if I had been plunged into the depths of grief.

### Performance of models

Table 4 shows the performance parameters of the constructed models. In all modeling procedures, precision tended to be high for "physical" and "work/financial" and low for "treatment". Although inferior to "physical", the precision of the other labels, “psychological” and “family/friends” was in the neighborhood of 0.60~0.70. Fine-tuning alone showed little impact on model performance, due to differences in data sources. The model with domain adaptation by MLM added had the highest precision for the LifePalette data. When both DIPEx and LifePalette data were used, domain adaptation tended to have little effect on performance. Overall, however, the models with MLM domain adaptation performed better in terms of precision, F1 score, and exact match accuracy. In particular, the performance of extracting the "treatment" label was improved after domain adaptation. Accuracy and recall were almost unchanged before and after domain adaptation. In terms of statistical significance, the precision of the model applying MLM using LifePalette was higher than that of the baseline model for the "treatment" and "physical" labels (p < 0.05). In addition, for the "work/financial" label, the precision for the all models except the DIPEx raw model and DIPEx (P)+LifePalette model was higher than that of the baseline model (p < 0.05).

**Table 4:** Performance of models.

Model	Labels																						
	Treatment				Physical				Psychological				Work/financial				Family/friends				Exact match	Ave F1	
	Acc	Pre	Rec	F1	Acc	Pre	Rec	F1	Acc	Pre	Rec	F1	Acc	Pre	Rec	F1	Acc	Pre	Rec	F1			
**Fine-tuning only**																							
**Baseline** (LifePalette)	0.80	0.24	0.22	0.23	0.80	0.78	0.62	0.69	0.79	0.40	0.57	0.47	0.93	0.59	0.57	0.57	0.89	0.59	**0.70**	**0.64**	0.44		0.52
DIPEx (P)	0.85	0.25	0.11	0.15	0.79	0.75	0.76	0.75	0.86	0.61	0.41	0.47	**0.96**	0.88	0.57	0.68	0.89	0.61	0.43	0.49	0.49		0.51
DIPEx (P)+LifePalette	0.84	0.31	0.17	0.20	0.80	0.76	**0.81**	**0.78**	0.84	0.53	**0.59**	**0.54**	**0.96**	0.85^**†**^	**0.68**	0.74	0.89	0.64	0.61	0.62	0.47		**0.57**
**Fine-tuning** ^*^ **+ MLM**																							
DIPEx raw	0.85	0.40	**0.19**	0.24	0.80	0.80	0.72	0.74	0.86	0.65	0.49	**0.54**	0.95	0.83	0.59	0.69	**0.91**	0.68	0.58	0.62	0.52		**0.57**
DIPEx (P)	**0.87**	0.43	0.15	**0.22**	0.80	0.77	0.77	0.76	**0.88**	**0.70**	0.44	0.53	**0.96**	0.89^**†**^	0.61	0.71	**0.91**	0.71	0.52	0.59	**0.55**		**0.57**
LifePalette	**0.87**	**0.51** ^**†**^	0.10	0.16	**0.82**	**0.82** ^**†**^	0.73	0.77	0.86	0.62	0.45	0.51	0.95	**0.90** ^**†**^	0.45	0.58	0.90	**0.73**	0.49	0.57	**0.55**		0.52
DIPEx (P)+LifePalette	0.85	0.26	0.13	0.16	**0.82**	0.78	0.80	**0.78**	0.87	0.69	0.45	0.52	**0.96**	0.89^**†**^	**0.68**	**0.76**	0.90	0.66	0.52	0.56	0.52		0.56

This table details the evaluation results for each concern label. "Acc," "Pre," "Rec," and "F1" represent accuracy, precision, recall, and F1 scores." Exact match" indicates the exact match accuracy of all labels and "Ave F1" indicates the average F1 value of all labels. As a baseline, we show the results of testing the model in our previous study (Watanabe et al. [24]) with the DIPEx data. The three ’fine-tuning only’ models show the performance of a baseline model using LifePalette (blog post data) and a J-based BERT model fine-tuned using only DIPEx (P) or DIPEx (P) + LifePalette (as additional data).

In the "Fine-tuning + MLM" method, domain adaptation was performed before fine-tuning with DIPEx (P) (model as * in the table), and four datasets were tested: DIPEx raw (unprocessed interview data), DIPEx (P), LifePalette, and DIPEx (P)+LifePalette.

The dagger marks indicate a statistically significance difference in the precision of the extraction of the “treatment”, "physical" and "work/financial " labels between the evaluated models after Bonferroni correction; † p < 0.05 for Baseline (LifePalette) versus other models.

## Discussion

### Principal findings

Our results show that developed multi-label classifier can deal with multiple concerns. Among the five targeted concerns from, the extraction performance was particularly high with regard to work, household finances, and physical concerns. Also, the proposed method of domain adaption using MLM provides better performance for multi-label classification in the patients’ narratives domain compared to the approach using only fine-tuning. In particular, the model applying MLM with LifePalette had a significantly higher precision for “treatment” and “physical” labels. Importantly, these concerns can be extracted from text based on Japanese conversation, which includes a wide variety of complex mispronunciations and onomatopoeia. This suggests that our approach could be a step forward in the development of future systems for providing appropriate information to cancer patients.

### Analysis of model performances

An error analysis was conducted to validate the performance of each label assignment. Concerning the labels "physical" and "work/financial," we considered that the text terms assigned to these labels are easily comprehensible and discriminable for the model. Specifically, if descriptions such as "pain," "nausea," or "feeling uncomfortable" were present, the label "physical" was assigned with a high probability, while if descriptions such as "money," "insurance," or "return to work" were present, the label "work/financial" was assigned with a high probability. For the "physical" label, many descriptions focused on specific body parts, and included adjectives such as "terrible" to describe the cancer status, "shock-like sensations," and "mastectomy." These descriptions were often accompanied by psychological shock and distrust of the hospital attended. Referring to the number of times the labels co-occurred, the top multi-labeled combinations were "physical" and " psychological " and "physical" and "treatment", with these two combinations accounting for approximately 60% of the total.

Conversely, for "work/financial," there were descriptions such as "interpersonal relationships at work" and "discussions about the cost of treatments." These expressions are specific to this concern and are less related to other concerns than the descriptions about the body, which are more common in "physical". As for the labeling results, "work/financial" alone accounted for around 70% of the results. For these reasons, it is considered that, although the number of labels for "work/financial" was far fewer than for "physical", it may have shown the same or better extraction performance than "physical". Nevertheless, the classification models may not fully distinguish whether or not these concerns are cancer-related.

As for the other three labels, they all account for nearly 15% of all labels granted, but there are marked differences in extraction performance. Among them, that of “treatment” was particularly low. There is no significant difference in the percentage of each label given on its own, and if multiple labels were given, the majority of the cases in which any of the labels were given were in combination with "physical".

For the "treatment" label, we believe that while specific patterns of concern existed, they varied widely and were difficult for the model to learn due to the small data population. Furthermore, since medical treatment-related terms are present in most texts, it is difficult to classify "treatment" labels by phrase, and the presence or absence of concerns must be determined from the context. With respect to the " psychological " label, labels were correctly assigned to texts with negative emotion words such as "shock" and "anxiety," but labels were misassigned to expressions that counteracted negative emotions. These expressions represent overcoming the emotional damage caused by cancer, but the model did not seem to understand this point. For the “family/friends” labels, labels were assigned to texts in which words such as “parents” and "siblings” were used to describe family members. However, labels were also given to positive statements such as “my family helped me to continue my cancer treatment”, so it is presumed that the learning is still not sufficient.

The application of domain adaptation resulted in an overall improvement in model performance, particularly in the precision of "treatment", which was notably low in the original model, although recall was not improved (Table 4). We speculate that this was due to successful learning of commonalities between the DIPEx and LifePalette datasets, leading to improved precision. Indeed, upon examination of the texts in which the model correctly classified “treatment”, it was observed that in many cases, the model correctly classified concerns also present in the LifePalette dataset, but was unable to correctly classify a diverse range of concerns unique to the DIPEx dataset. These results provide valuable insights into the reasons for the lack of improvement in recall.

### Comparison with prior studies

In a previous study [24], on which the current study is based, a classifier created from blogs had the highest prediction precision for "physical" at over 0.80, while the prediction precision for other concern labels was around 0.60. Although the model created in this study was based on interview data, the extraction performance for "physical" was as good as in the previous study. The performance of "work/financial" was superior to that of previous studies, whereas the performance of "treatment" was inferior. However, the domain adaptation improved precision for this label by an average of more than 0.15. So far, Watanabe et al.’s study is the only one that has utilized leading-edge transformer-based NLP methods, such as BERT, for dealing with patients’ multiple concerns. Most studies have used classical algorithms, such as SVM and NB, and primarily focused on sentiment analysis [14, 37]. Studies using such methods, while the models are lightweight, show limitations in dealing with the domain of patients’ words [38, 39]. In this study, we also built a classical algorithmic model, but it failed to reach the classification performance of the deep learning model (S3 Table). Therefore, it is appropriate to utilise models such as BERT, which can take into account the context of the patient’s narrative. There have been studies using BERT for text related to breast cancer, with each study exploring ways to improve the performance of the model. However, there are no previous studies on domain adaptation using MLMs in the domain of patient narratives [40–42]. Although there is no previous research on this method conducted in the area of patient narratives, there are examples of improved model performance by making use of specific domain data resources for both English and Japanese texts [43, 44]. In this study, as in previous studies, MLM improved the performance of models.

### Limitations

This study was subject to several limitations.

First, the dataset used in this experiment is small and imbalanced. In general, approximately 500 texts may not be sufficient for building machine learning models for text classification. However, in studies such as this, dealing with medical data containing personal information, datasets are often small, and problems such as bias in patient background factors are difficult to avoid. We attempted to mitigate this problem by having the model learn data from the relevant domain through domain adaptation. In fact, the dataset contained approximately 200 "physical" labels, which was the highest number of assigned labels, and approximately 40 "work and financial" labels, which was the lowest number of assigned labels, and this may have affected the model’s learning results.

Second, this model has the limitation that only 512 words can be input. Texts exceeding 512 words amounted to less than 30% of the dataset, and the omitted text was only a few dozen words, so there was little information loss. We tried methods that varied the input limit of 512 words and methods that used only data with less than 512 words, but the model performance showed little change.

Third, the data set used in this study includes only text data transmitted by patients. Thus, although there are many different types of breast cancer, and the progression of breast cancer varies from person to person, which may lead to significant differences in mental status and living environment, it is not possible to take account of patients’ precise treatment status in this study.

Fourth, although this study focuses on the usefulness of using unprocessed interview transcripts for training, there is a risk that too much wordiness will be learned if too much unstructured data is used for training. Therefore, minimal processing would likely need to be applied to avoid this effect, but there is no clear definition of the type of unspokenness or which state “unprocessed” refers to with respect to interviews, so it is difficult to identify an appropriate processing method. This study does not address this issue.

### Future work

The concern classifier developed here is superior in extracting "physical" and "work/financial" concerns and could be applied to patient support systems to facilitate the identification of patients suffering from side effects or in need of work/financial support. From the viewpoint of patient support, it would be desirable in the future to provide more personalized information by combining this text-based system with other patient data, such as the patient’s stage of treatment. In addition, given the effectiveness of the NLP model for spoken text in this study, it could be applied to systems that extract patients’ needs and concerns from their voice input. Patient communications, such as blogs and interviews, as handled in this study, have high barriers to entry for patients, and it takes time for the information to become available to the public. Thus, it would also be desirable to develop systems that allow patients to communicate their needs and concerns directly to medical professionals from their homes, and to build chatbots that can automatically respond to such communications and provide information. We hope that the challenge of adapting the NLP model to spoken language, as in this study, will be the first step in the development of such a system.

## Conclusions

In this study, we tested whether the method for extracting concerns from patient-generated text proposed in a previous study is also effective for interview transcriptions. Our results suggest that it is possible to effectively extract the "physical" and "work/financial" concerns of breast cancer patients using BERT-based methods, even for spoken text such as interview transcriptions. We also found that domain adaptation using MLM is effective for improving performance.

## Supporting information

S1 Table Annotation guidelines. (DOCX)

S2 Table Table 3, which also includes the original texts (Japanese). (DOCX)

S3 Table The performance of the classifiers based on classical algorithms. (DOCX)
