# Correction: Exploring a method for extracting concerns of multiple breast cancer patients in the domain of patient narratives using BERT and its optimization by domain adaptation using masked language modeling

**DOI:** 10.1371/journal.pone.0329664

**Published:** 2025-08-01

**Authors:** Satoshi Watabe, Tomomi Watanabe, Shuntaro Yada, Eiji Aramaki, Hiroshi Yajima, Hayato Kizaki, Satoko Hori

The phrase “concerns of multiple” is incorrect in the article title. The correct title is: Exploring a method for extracting multiple concerns of breast cancer patients in the domain of patient narratives using BERT and its optimization by domain adaptation using masked language modeling. The correct citation is: Watabe S, Watanabe T, Yada S, Aramaki E, Yajima H, Kizaki H, et al. (2024) Exploring a method for extracting multiple concerns of breast cancer patients in the domain of patient narratives using BERT and its optimization by domain adaptation using masked language modeling. PLoS ONE 19 (9): e0305496. https://doi.org/10.1371/journal.pone.0305496.

## References

[1] Watabe S, Watanabe T, Yada S, Aramaki E, Yajima H, Kizaki H, et al. Exploring a method for extracting concerns of multiple breast cancer patients in the domain of patient narratives using BERT and its optimization by domain adaptation using masked language modeling. PLoS One. 2024;19(9):e0305496. doi: 10.1371/journal.pone.0305496 39241041 PMC11379386

