# Supplementary material for: Exploring a method for extracting concerns of multiple breast cancer patients in the domain of patient narratives using BERT and its optimization by domain adaptation using masked language modeling
Source: PLoS One. 2024 Sep 6;19(9):e0305496. doi: 10.1371/journal.pone.0305496 (PMC11379386; doi:10.1371/journal.pone.0305496)
Supplement: S1 Table — (DOCX) [file pone.0305496.s001.docx]

**S1 Table. Annotation guidelines**

| Label | Detail |
| --- | --- |
| Treatment | - Being jealous of healthy visitors and pregnant women - I want to stay out of the hospital, but I can't - Worries about postponing treatment - Anxiety about the end of treatment - Concerns about:   Outpatient, hospitalization, discharge, and transfer   - Hospital response, facilities, and relationships with medical personnel - Notification, informed consent, and second opinions - Diagnosis and treatment - Medical treatment - Not being able to take a clinical trial - Clinical trial selection - Response of the local hospital after discharge - Postponing treatment |
| Physical | - Concerns about cancer symptoms, side effects of medication, and after-effects of treatment   (Label any side effects if they occur.)  (Also label hair loss, loss of fertility, etc.)   - Fever, malaise, etc. - Abnormal values - Insomnia - Physical pain due to medical treatment |
| Psychological | - Vague anxiety about cancer - Anxiety about recurrence - Depression, despair, shock, sadness, fear |
| Work/financial | - Worries about high medical costs, work, and coworkers - The burden of cancer on work |
| Family/friends | - Worries about family, loved ones, children, and local community - I take it out on my family. - Having a hard time being treated as a sick person by others - Feeling guilty about them - Worries about housework - Worries about pregnancy and partner |
