# Supplementary material for: Exploring a method for extracting concerns of multiple breast cancer patients in the domain of patient narratives using BERT and its optimization by domain adaptation using masked language modeling
Source: PLoS One. 2024 Sep 6;19(9):e0305496. doi: 10.1371/journal.pone.0305496 (PMC11379386; doi:10.1371/journal.pone.0305496)
Supplement: S2 Table — (DOCX) [file pone.0305496.s002.docx]

**S2 Table. Table 3, which also includes the original texts (Japanese)**

| **Labels** | **Texts** |
| --- | --- |
| Treatment | 再建の仕方はちょっと、悩みまして。あの放射線治療を受けていたので、放射線治療を受けた人が人工物で再建するのは、皮膚が伸びにくいから難しいといわれていて。  (I was a little apprehensive about the breast reconstruction procedure. I was told that because I had undergone radiation therapy, it would be difficult to reconstruct my breast with an artificial one because the skin would not stretch easily.) |
| Physical | 爪に症状が出てきて、爪が真っ黒く変色したりとか、あと、爪に少しでも物が当たると激痛が走る感じで。  (My fingernails started showing symptoms. The nails turned black, and even the slightest object hitting them caused severe pain.) |
| Psychological | 第1回目のときには、もうね、私、随分ね、泣きましたけどもね。「まあ、私ね、もうこのまま死んじゃったら、この後、どうなるんだろう」と思いましたよね。  (I cried a lot during the first cancer announcement and I thought, "What will happen to the rest of my family after I die?") |
| Work/financial | しかし心配なのはやっぱりお金かなあ（笑）今はまだ何とかやっているけど、毎週2万5千円が飛んでいく。  (But I guess what worries me is the money (laughs). I'm still getting by now, but every week 25,000 yen goes flying by.) |
| Family/friends | 子どものこととか、主人のこととかね。うちのことはね。どういうふうになるかと思って。本当にもう、何ていうか、自分でもね、もうどうしようもできない、こう、あれですね。悲しみのどん底に、陥れられたっていう感じでしたね。ええ。  (I wondered what would happen to my children and my husband. I really had no choice, I couldn't help myself. I felt as if I had been plunged into the depths of grief.) |
