# Supplementary material for: Exploring a method for extracting concerns of multiple breast cancer patients in the domain of patient narratives using BERT and its optimization by domain adaptation using masked language modeling
Source: PLoS One. 2024 Sep 6;19(9):e0305496. doi: 10.1371/journal.pone.0305496 (PMC11379386; doi:10.1371/journal.pone.0305496)
Supplement: S3 Table — (DOCX) [file pone.0305496.s003.docx]

**S3 Table. The performance of the classifiers based on classical algorithms.**

Model performance of bag of words

| **Label** | **Accuracy** | **Precision (SD)** | **Recall (SD)** | **F-score (SD)** |
| --- | --- | --- | --- | --- |
| Treatment | 0.87 | 0.00 | 0.00 | 0.00 |
| Physical | 0.74 | 0.71 | 0.67 | 0.68 |
| Psychological | 0.83 | 0.41 | 0.24 | 0.29 |
| Work/financial | 0.91 | 0.33 | 0.17 | 0.21 |
| Family/friends | 0.86 | 0.49 | 0.40 | 0.44 |
| Macro average | 0.84 | 0.40 | 0.30 | 0.33 |

Model performance of word2vec

| **Label** | **Accuracy** | **Precision (SD)** | **Recall (SD)** | **F-score (SD)** |
| --- | --- | --- | --- | --- |
| Treatment | 0.83 | 0.22 | 0.31 | 0.25 |
| Physical | 0.73 | 0.72 | 0.58 | 0.64 |
| Psychological | 0.81 | 0.38 | 0.32 | 0.34 |
| Work/financial | 0.93 | 0.67 | 0.40 | 0.48 |
| Family/friends | 0.87 | 0.55 | 0.48 | 0.51 |
| Macro average | 0.84 | 0.51 | 0.42 | 0.44 |
